# Solubility and Metastability of the Amyloidogenic Core of Tau

**DOI:** 10.1021/acschemneuro.5c00784

**Published:** 2026-01-23

**Authors:** Emil Axell, Andreas Carlsson, Max Lindberg, Katja Bernfur, Emma Sparr, Sara Linse

**Affiliations:** † Biochemistry and Structural Biology, Lund University, SE-221 00 Lund, Sweden; ‡ Division of Physical Chemistry, Department of Chemistry, Lund University, SE-221 00 Lund, Sweden

**Keywords:** amyloid solubility, monomer−fibril equilibrium, solubility limit, metastability, tau amyloid core, protein quantification

## Abstract

Intracellular deposits of neurofibrillary tau tangles and extracellular Aβ plaques are closely associated with Alzheimer’s disease. The mapping of thermodynamic parameters, including solubility limits, indicates when a protein forms amyloid fibrils or remains in solution. This reveals the direction of change of the system and may help in understanding drift and steady states in living systems. Here we have developed methodology for tau solubility quantification and determined the solubility of the amyloidogenic core fragment of tau *in vitro*. We monitored the concentration of free tau304–380C322S fragment at 37 °C in phosphate buffer at pH 8.0 using three separate methods: HPLC-UV, derivatization with ortho-phthalaldehyde and scintillation counting. The measurements were repeated over time until a stable value was reached, implying that an equilibrium with fibrils had been established. The solubility measurements converged on a free monomer concentration of 6.1 ± 3.5 nM, which represents the solubility of the fragment under the current experimental conditions.

## Graphical abstract

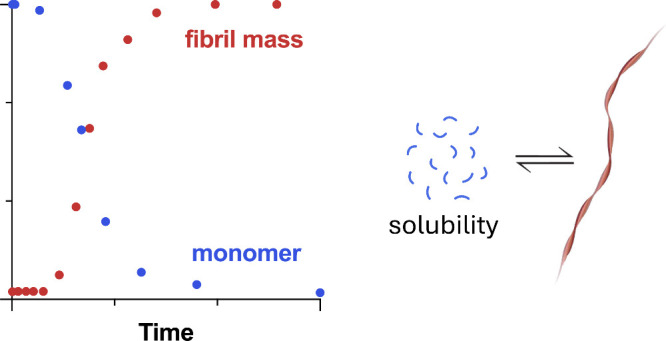

## Introduction

The self-assembly of proteins and the subsequent accumulation of amyloid fibril deposits are closely associated with multiple diseases and affect various organs of the human body. In the brain, deposits of the amyloid β peptide (Aβ) and the tau protein make up the characteristic plaques and neurofibrillary tangles observed in the most common form of dementia, Alzheimer’s disease (AD). The disease inflicts immense individual suffering, and the societal burden is substantial. The accumulation and deposition of tau aggregates are also a characteristic feature of several other neurodegenerative disorders collectively referred to as tauopathies.^1^ The involvement of tau amyloid accumulation in neurodegenerative diseases calls for physicochemical characterization of all aspects of tau aggregation including its equilibrium and kinetics.

Due to the high apparent solubility of recombinant full-length tau and its apparently high barriers toward aggregation *in vitro*, additional chemical additives or surfaces can be introduced in order to induce aggregation. Many of the molecules that have been shown to trigger tau aggregation are polyanions like heparin and RNA, or negatively charged fatty acids.^2–5^ Structure elucidation through cryo-TEM has revealed that tau fibrils formed in the presence of heparin display a different morphology than that of tau fibrils found in brains of patients suffering from AD or other tauopathies.^6^ The lack of reliable model systems to study tau aggregation in the absence of external inducers has hampered the field. Toward this end, we have previously developed a protocol for the expression and purification of a fragment of tau, which spans the amyloidogenic core region of *ex vivo* tau fibrils from the AD brain.^7^ The fragment comprises amino acid residues 304–380 according to 2N4R (also called tau441) numbering and forms fibrils in low ionic strength buffer solution without external inducers.^8^ Cysteine 322 was mutated to serine to enable the fragment to be studied under nonreducing conditions without forming artificial dimers. The present study thus employs the same fragment, tau304–380C322S, herein termed tau or tau AD core fragment.

We recently developed an affordable high-throughput protein solubility assay.^9^ In the present work, we demonstrate the use of this assay for the tau AD core fragment and further develop additional quantification strategies using radiolabeled peptide, enabling quantification in more complex sample matrices. Furthermore, we introduce an HPLC-UV/MS method for quantification and discrimination between modified peptides and the original starting material.

## Results and Discussion

First, we present a methodology to determine the solubility of the tau AD core fragment, using centrifugation as the separation method and HPLC-UV absorbance for quantification and mass spectrometry (MS) for identification. Then, we investigate and discuss how the choices of the methodology and quantification techniques may influence the results.

There are several difficulties in measuring the solubility of an amyloidogenic peptide. We have identified three main hurdles that need to be overcome for reliable measurements and demonstrate the advantages and disadvantages with different methodologies.

*(I) Can the equilibrium state be reached in a reproducible manner?* It is important to maintain a homogeneous dispersion during an aggregation process. If fibrils sediment from solution, these fibrils are less capable of catalyzing further aggregation compared to the dispersed state where fibrils and monomers coexist at the same proportions in every part of the sample. In a situation with sedimenting fibrils, metastability will be sustained by the primary nucleation energy barrier, which is much higher than the elongation and secondary nucleation energy barriers.^10^ In addition, we observe that the peptide undergoes changes in its primary structure during prolonged incubation such as chemical modifications and/or truncations. This is a common phenomenon in solutions of unfolded proteins when incubated at 37 °C for long periods of time, and has been classically explored in limited proteolysis to determine the organization of protein domains.^11^ Hence, to maintain an intact peptide for reliable solubility measurements, it is necessary to reach equilibrium between the monomer and fibril within a reasonable laboratory time frame to avoid degradation of the peptide. Here, we used stirring with a magnetic stir bar to keep the fibrils in dispersion and, thus, speed up the aggregation process dramatically. The importance of stirring during fibril formation and the notable metastability of tau under idle conditions is further explored in the SI (Figures S1–S3). Interestingly, we observe degradation into more distinct species, with different retention times, when analyzed by HPLC, in the samples that are initially idle and then stirred (Figure S3), compared to those that were idle or stirred all the time. This may be explained by the fibril surface catalyzing chemical reactions, a phenomenon that has been reported for other amyloid peptides.^12,13^

*(II) Can fibrils be efficiently separated from monomers?* In methods that detect all proteins in a sample, the removal of fibrils is necessary for quantification of the monomer concentration. All techniques used in this work are based on a separation step, making its performance an important factor to investigate. Here, we use filtration as well as centrifugation, and evaluate the advantages and disadvantages of both methods.

*(III) Can the concentration of peptides be reliably quantified?* Depending on sample complexity, concentration of interest, and signal parameters available for detection in a certain system, various techniques may be more or less suitable. Tau has a solubility in the nanomolar range, which greatly reduces the number of possible techniques. We demonstrate here that analytical HPLC with UV absorbance detection, using reversed phase chromatography coupled to a mass spectrometer, is a reliable way to quantify such low concentrations of tau peptide in solution and distinguish between peptide variants (modifications and/or truncations) and impurities. We compare this method to quantification using ortophthalaldehyde fluorescence (OPA)^9^ and liquid scintillation counting (LSC), where the former is quick, easy and affordable but lacks selectivity and sensitivity compared to HPLC-UV and the latter is an option for samples with higher component complexity and relies on radioisotope labeling of the tau AD core fragment.

### The Solubility of Tau

The solubility of the tau AD core fragment was investigated by examining its concentration in solution during the aggregation process, starting from supersaturated solutions at two monomer concentrations (5 and 10 μM) and approaching equilibrium (Figure 1(a)). Monomers were separated from fibrils by centrifugation, and the tau concentration in the supernatant was quantified by HPLC-UV absorbance (Figure 1(b), (d)). Using HPLC-MS, the A_280_ and A_205_ peaks at 3.6 min were identified as intact tau (Figure S4). When the area of this peak was integrated at different time points during the course of the aggregation process, the free monomer concentration was found to plateau after 16 h at 6.1 ± 3.5 nM (n = 10), consistent with convergence toward the solubility limit. The concentration of free monomers at equilibrium becomes constant because the chemical potential of the monomers in solution and the monomers in the fibril phase are equal, resulting in a defined solubility limit.^14,15^ We refer to this plateau as the apparent solubility, because experimental measurements cannot distinguish whether the system has reached the true thermodynamic equilibrium or remains in a metastable (kinetically trapped) state. Another challenge arises when chemically modified or degraded tau species are present, as they may alter the measured monomer concentration either if they form coaggregates with intact peptides or if they themselves have a different solubility. Another peak in the chromatograms, i.e. a species eluting before the tau monomer at ∼ 3.3 min (Figure 1(b)) is present in all samples from the beginning. This species does not appear to be incorporated into the fibrils but remains in solution after equilibrium has been reached. It represents a small but detectable trace contaminant, with a peak area (UV_205_) less than 1/1000 of the initial monomer peak. The peak does not have any absorbance at 280 nm or a prominent mass in the mass spectrum. Therefore, we conclude that this peak represent a minor contaminant (<0.1%) and should not be analyzed in the context of the formation of tau fibrils.

**Figure 1:**
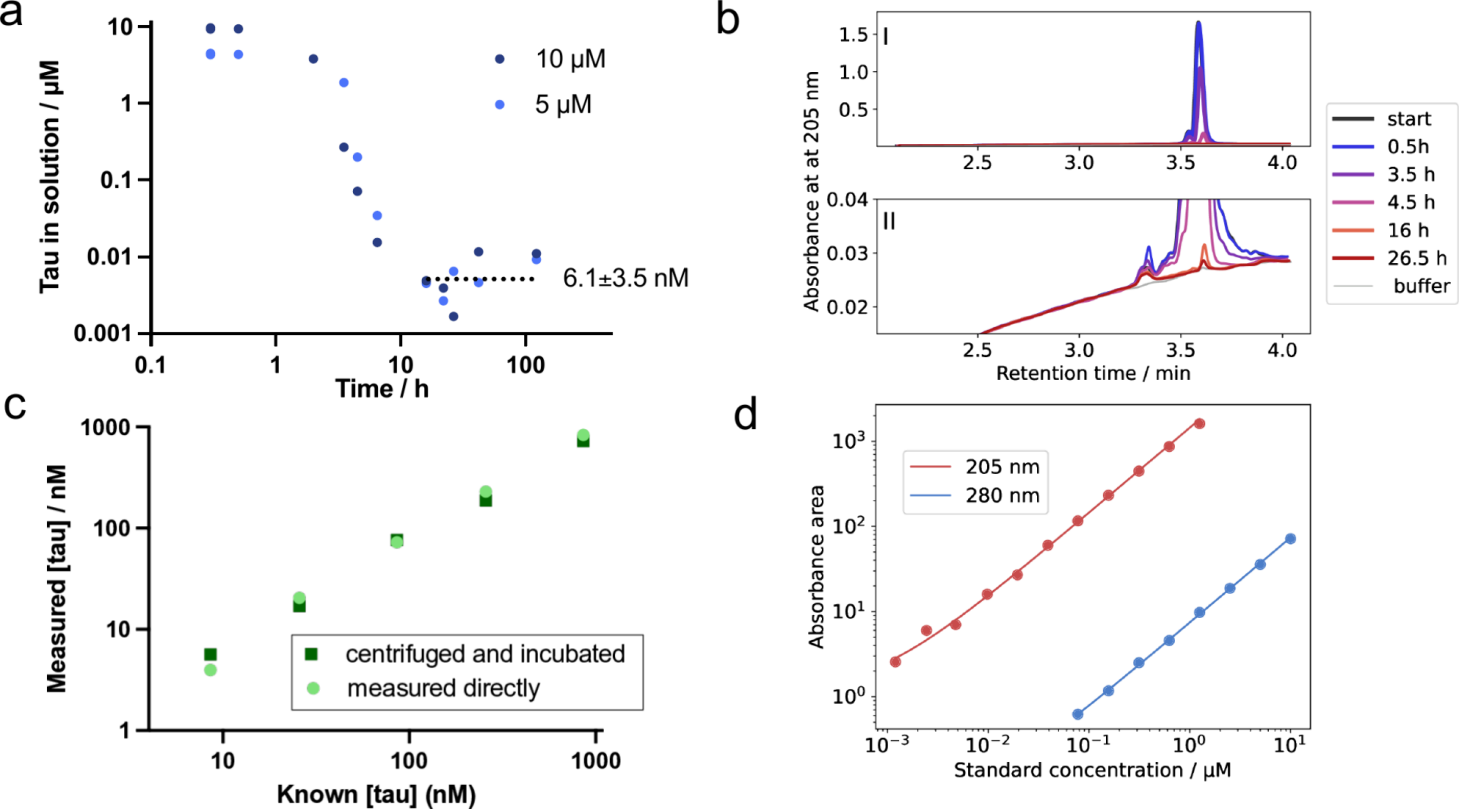
Approach of the tau aggregation equilibrium accelerated by stirring. (a) Concentration of tau in solution during fibril formation, starting from 10 μM (dark blue) and 5 μM (light blue) tau monomers in 20 mM sodium phosphate, 0.2 mM EDTA, 0.02% NaN_3_, pH 8.0 at 37 °C, after removal of fibrils by centrifugation at 20000 x*g* for 30 min. The obtained concentrations after 16–122 h were used to calculate a mean and standard deviation of 6.1 ± 3.5 nM (*n* = 10). (b) Chromatograms monitored by the absorbance at 205 nm of the samples with 5 μM start concentration, at time points as given by the legend. The peak at 3.6 mL was identified as intact tau304–380C322S by mass spectrometry, and it is the area of this peak that is used to calculate the values given in (a). (c) Evaluation of protein losses. Known tau concentrations were either directly injected in the HPLC (light green circles) or subject to the same surface exposure and centrifugation steps as the samples in (a) before measured with HPLC (dark green squares). (d) The standard curves used to calculate concentrations from the areas of the chromatograms. Above 1 μM, the standard curve based on the absorbance at 280 nm was used, and below the absorbance at 205 nm was used.

When working with proteins at concentrations in the nanomolar range, it is important to consider potential but unintentional protein losses due to adsorption to surfaces, such as sample tubes and instrument tubing, during sample preparation and quantification. As a control, samples of known concentrations were injected directly into the HPLC instrument or first subjected to all experimental surfaces, such as microfuge tubes and 96-well plates (Figure 1(c)). Based on these control experiments, we conclude that protein losses due to adsorption are negligible compared to dilution errors, validating 6.1 ± 3.5 nM as a measure of tau solubility under current conditions.

### Separation Method: Filtration and Centrifugation

The efficacy of monomer separation from fibrils was examined for filtration and centrifugation by quantifying the remaining species in the filtrate or supernatant, respectively (Figure 2a). Quantification of the remaining monomers was done through derivatization with the primary amine reactive dye, OPA. In parallel, the growing fibril fraction was monitored in a separate reaction mixture supplemented with 10 μM ThT, starting from 10 μM monomers, from which 50 μL was withdrawn at each time point after which the fluorescence intensity (F.I) was measured (Ex_448_-Em_480_), (Figure 2e). To rule out the loss of monomer to the filter, tau monomers in a concentration range were derivatized with OPA with or without filtration (Figure 2b). Negligible monomer adsorption was observed. However, trace contaminants from the filters contributed a minimal fluorescence background. To investigate the potential effect of this background on the final solubility measurement, a sample with a known concentration at the lower end of the standard curve was reacted with OPA with or without filtration. The same was done for the pure buffer (Figure 2c). The minimal, yet detectable background introduced by filtering the samples was found to be smaller than the standard deviation of the experimental method. To further investigate filtration as an effective strategy for separating monomers from fibrils, the fluorescence intensity of tau fibrils was measured in the presence of the amyloid reactive Congo Red derivative X34, before and after filtration (Figure 2d). The filters effectively removed all X34-binding species, and negligible X34 fluorescence intensity was detected in the filtrate, suggesting that fibrils had been effectively removed from the solution. In conclusion, both filtration and centrifugation effectively separate monomers from fibrils, yielding comparable solubility measurements.

**Figure 2:**
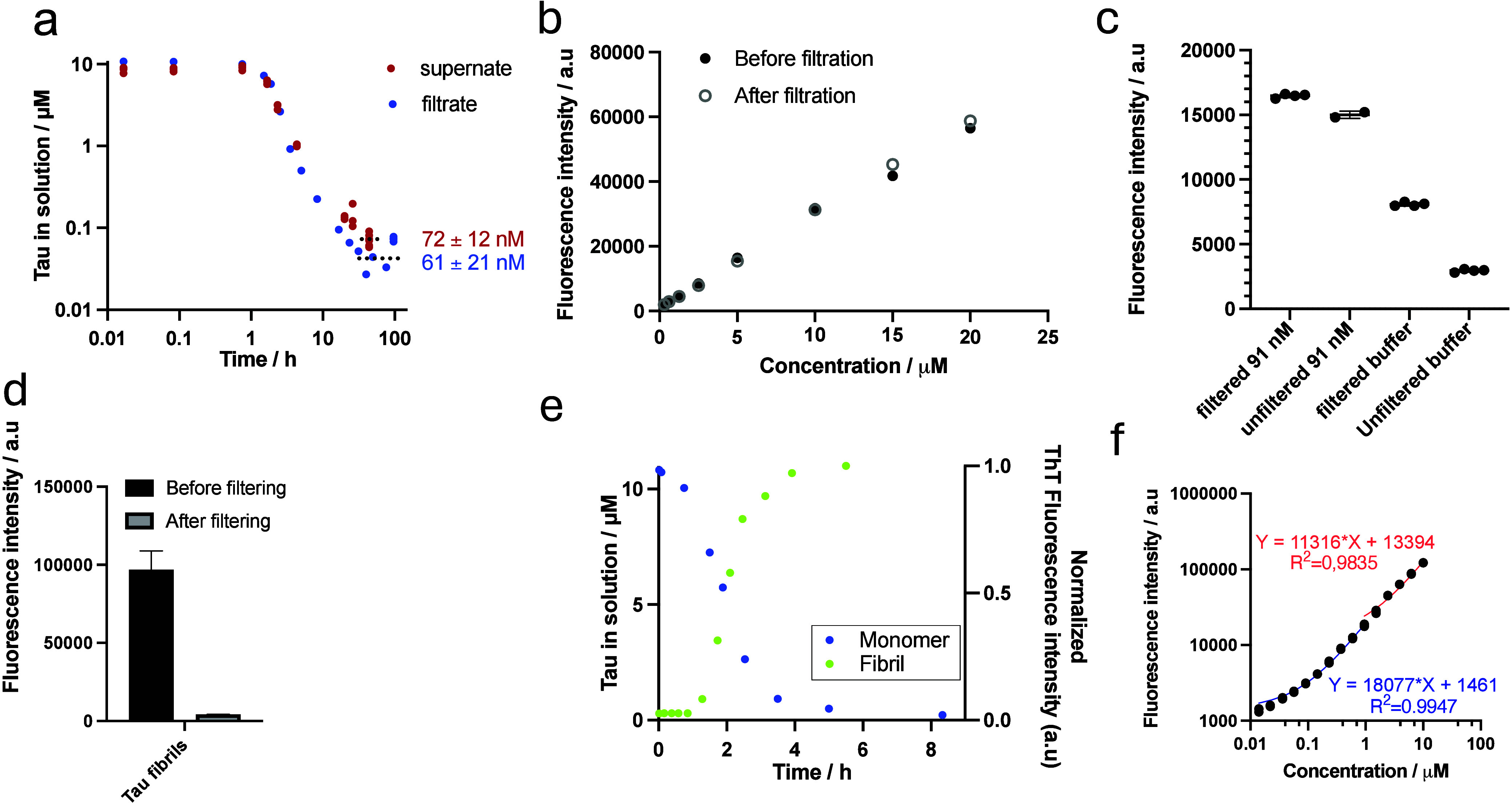
Approach of the tau aggregation equilibrium as a function of time under stirring at 37 °C in 20 mM sodium phosphate, 0.2 mM EDTA, 0.02% NaN_3_ at pH 8. (a) Remaining tau in solutions quantified with OPA after separation from fibrils by filtration (blue) or centrifugation (red), visualized on log_10_ axes. (b) To rule out loss of monomers to the filter, OPA fluorescence intensity (F.I.) of tau monomers over a concentration range was measured before and after filtration. (c) To investigate the background F.I. from a compound released by the filter, the OPA F.I. was measured before and after filtration of 91 nM tau monomer and buffer. (d) In order to evaluate the effective separation of monomers from fibrils by filtration, the amyloid binding dye X34 F.I. of tau fibrils was measured before and after filtration. (e) The increasing fibril fraction was followed in parallel with the decreasing monomer fraction, on the right *y*-axis: normalized F.I. of 10 μM tau monomers followed as a function of time with ThT fluorescence (green); left *y*-axis same as blue data in (a) but visualized on a linear axis. (f) Standard curves used to quantify the filtrate concentrations in (a) on Log_10_ axes; two segments of a dilution series were used, one above, and one below 1 μM to ensure linearity.

### Comparing Quantification Methods: HPLC, OPA-Fluorescence, and LSC

Having established that HPLC can distinguish between intact tau and modified variants of the peptide, we now ask whether a more crude quantification strategy using OPA may be a sufficient method for the determination of the total concentration of species in suspension. Furthermore, in order to quantify a low concentration in a sample with high component complexity without perturbing the system with a fluorescent probe or using separation by HPLC, liquid scintillation counting (LSC) is an option. To compare the latter technique with HPLC and the fluorescence of OPA, tau was radiolabeled through expression in minimal medium with tritiated glucose added just prior to the induction of protein expression. A solution of 10 μM monomers was incubated at 37 °C with stirring and fractions were removed during the aggregation process, to be filtered and quantified using LSC. The tau concentration was obtained using a standard curve, as described in the SI (Figure S9). The resulting concentrations are shown in Figure 3, and to allow comparison with HPLC and OPA as quantification techniques.

**Figure 3:**
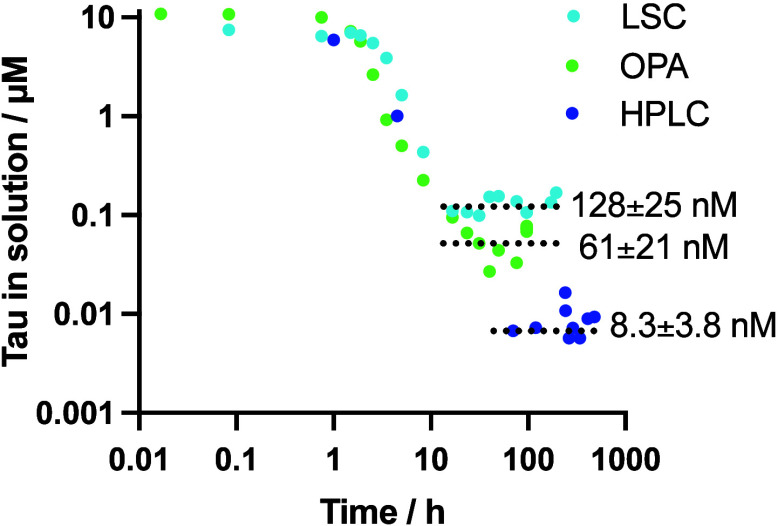
Three different quantification techniques to probe the concentration of tau in solution during aggregation from 10 μM monomers after removal from fibrils by filtration. The OPA data series is replotted from Figure 2(a), and the HPLC data are replotted from Figure S1­(b).

It should be noted that the concentrations obtained from the OPA fluorescence and LSC are both close to their quantification limits, as seen from the standard curves (Figures 2 and S9), to which the factor two difference between them can be attributed. Labeling with a more radioactive isotope or larger amounts of tritiated glucose during expression could potentially improve the limit of detection. Another important aspect to bear in mind when comparing the values obtained with different quantification methods is that OPA fluorescence and LSC quantify total protein or total tau, respectively, and therefore, they reflect the combined concentration of all species in solution. In contrast, HPLC separates species and provides discrete concentration estimates on individual peak integrals. It is therefore the concentration obtained by summing up all the integrals in the HPLC chromatogram that should be compared to the values obtained by the other techniques. When this comparison is made for the chromatograms in Figure S1­(d), at 340 h, it sums to 128 nM and brings the different quantification strategies into good agreement with each other.

## Concluding Remarks

This work extends our previous amyloid protein solubility assay,^9^ introducing new methodologies for quantification and identification by HPLC-UV/MS with sensitivity in the low nanomolar range. We also introduce a strategy that enables quantification in more complex sample matrices, such as blood or cerebrospinal fluid, using LSC. Although this approach relies on radiolabeling of the peptide, it may provide an option in such cases to gain information on *in vivo* solubility. Next, we validate our previously developed OPA assay on tau, confirming its use as a rapid and affordable, albeit less selective method.

For systems with high nucleation barriers, such as tau, we demonstrate that controlled stirring effectively shortens the time spent in a metastable state. By accelerating fibril formation, this approach enables the equilibrium to be reached within laboratory time frames, making it possible to investigate the solubility of otherwise challenging systems. The quantification strategies employed in this work all rely on measuring the concentration of free monomers in solution, which requires an efficient separation step to remove fibrils. We compared filtration and centrifugation and found that both approaches effectively achieve this in our system, yielding consistent solubility values. Using these approaches, we determined the solubility of the amyloidogenic core fragment of tau to be ≈ 6 ± 3.5 nM under our experimental conditions. This places the current fragment of tau at the low end of the reported amyloid solubilities, substantially lower than Aβ40 ^9,14^ and α-synuclein^16^ and closer to the low nanomolar range than that of Aβ42.^17^ This emphasizes the strong thermodynamic driving force toward fibril formation of tau. At the same time, its aggregation *in vitro* is characterized by long lag phases, indicating high nucleation barriers. This combination of a strong thermodynamic driving force and a high kinetic barrier suggests that the sequence could have evolved internal kinetic protection against spontaneous aggregation. We could speculate about the potential implications in disease, where one could imagine that post-translational modifications, such as phosphorylation, may compromise this safeguard. Modifications can occur both before or after fibril formation and shift the balance toward aggregation by lowering nucleation barriers or generate species with extremely low solubility or high seeding capacity. Tau truncation products are abundant in the so-called ghost tangles which persist in the brain of individuals with AD after the neurons containing them have died.^18,19,20^ Our results suggest that such truncated fragments could potentially inherit the extreme insolubility of the core fragment investigated in this study, while also bypassing the kinetic protection of full length tau. The accumulation of short tau species creates a proteostasis dead end, which cellular clearance mechanisms cannot efficiently solubilize or degrade. Understanding this balance among solubility, metastability, and proteolysis may be key to future therapeutic strategies against tauopathies.

## Methods and Materials

### Expression and Purification

Tau304–380C322S (GSVQI​VYKPV​DLSKVT​SKSGS​LGNI​HHKP​GGGQ​VEVK​SEKL​DFKD​RVQS​KIGS​LDN​ITHVP​GGGNK​KIET​HKLT​FRE) was expressed and purified as previously described.^8^ The initial M was found to be removed in *E. coli* and thus not listed in the sequence. In brief, codons were optimized for *E. coli* expression and cloned in a Pet3a plasmid (purchased from Genscript, USA). The plasmid was transformed into *E. coli* (BL21 Star DE3 pLysS) from Invitrogen, USA. Overexpression was achieved through an autoinduction medium. Isotope enrichment of the tau fragment was achieved in minimal M9 medium with glucose supplemented with tritiated d-glucose from PerkinElmer as the main carbon source, detailed protocols can be found in Michaels 2020.^21^ In minimal medium, overexpression was induced with IPTG and the tritiated d-glucose was added 5 min before IPTG. Purification was done by boiling the supernatant after harvesting and breaking the cells by sonication. After boiling, the precipitated endogenous proteins were removed by centrifugation and the remaining soluble fraction was further purified by a series of ion exchange and size exclusion chromatography steps as described in detail in Rodriguez et al.^8^

The protein was stored lyophilized at −20 °C and prior to each experiment, subjected to a final size exclusion chromatography step on a Superdex75 10/300 column, and equilibrated in degassed and filtered experimental buffer. This was done to ensure monomeric and ultra pure protein as starting material. Absorbance was monitored at 280 nm and protein concentration was calculated by integrating the chromatogram and using the theoretical extinction coefficient of 1490 M^–1^ cm^–1^ estimated by Expasy protparam using the amino acid sequence.^22^

### Solubility Incubation and Separation of Soluble and Insoluble Species

Freshly isolated tau monomers were diluted to enable the apparent solubility to be reached from multiple concentrations. After dilution, monomer solutions were incubated to form fibrils in 5 mL protein LoBind 525–0792 Eppendorf tubes with stirring (200 rpm) using Teflon-coated magnetic stir bars (PTFE micro stir bar 8 × 1.5 mm VWR 442–0463). The samples were incubated at 37 °C to allow the supersaturated monomers to form fibrils and the apparent equilibrium to settle. After incubation, monomers were separated from fibrils by centrifugal filtering using acroprep 96 well filter plates with GHP membranes with pore size 0.2 μm (ref 8082, Pall corporation) at 2000 x*g* for 2 min or 20000 x*g* for 30 min in the HPLC data or ultra centrifugation at 100,000 x*g* for 1 h in an airfuge (Beckman Coulter) for the comparison of centrifugation and filtration using OPA in Figure 2(a).

### Monomer Quantification by o-Phthalaldehyde Fluorescence

After filtration or centrifugation, 50 μL of the monomer filtrate or supernatant was derivatized with 5 μL of the primary amine reactive dye o-phthalaldehyde (OPA, commercially ”Fluoraldehyde”, ref 26025, Thermo Fischer scientfic) and incubated for 10 min at room temperature. The reaction was carried out in black bottom 96-well plates (3686, Corning). Fluorescence intensity was measured using an excitation wavelength of 340 nm (30 nm slits) and emission wavelength of 440 nm (40 nm slits) with a dichroic mirror set at 387.5 nm in a plate reader (CLARIOstar, BMG), and gain and focal height were automatically adjusted. The unknown sample concentrations were calculated using the equations obtained from the unweighted linear regression of the fluorescent signal obtained from a minimum of six standard solutions of tau monomers, serially diluted from a known concentration calculated from integrating the FPLC chromatogram when isolating monomers.

### Monomer Quantification by HPLC-UV

For monomer quantification by HPLC-UV, 30 μL of sample was injected on a CN reversed phase column (BIOshell A160 Peptide CN column 66966-U, Sigma-Aldrich) with a Shimadzu Nexera X3 system connected to a single quadrupole mass spectrometer (LCMS-2020, Shimadzu) for peak identification; see SI (Figure S4). The column was kept at 60 °C and the sample was eluted with a linear gradient from 5 to 95% acetonitrile over 10 min in an aqueous mobile phase with 0.1% TFA flowing 0.5 mL/min. UV signal was collected at 205 and 280 nm simultaneously with a SPD-40 V detector operating at 10 Hz with “Standard” response and a cell temperature of 40 °C.

### Fibril Concentration As a Function of Time

The fibril mass was followed with stirring in a 5 mL tube in the presence of 10 μM ThT. The fluorescent intensity of 50 μL of fibril solution was measured at each time point in a BMG Fluostar omega with excitation at 448 nm and emission at 480 nm.

### Monomer Quantification by Liquid Scintillation Counting

Solubility incubations were performed as above but using ^3^H labeled tau. The samples were allowed to reach apparent equilibrium at 37 °C, and the soluble species were then separated from the fibrils by filtration or ultracentrifugation. Twenty μL of the filtrate was mixed with 3 mL Ultima Gold LLT scintillation liquid (PerkinElmer) and counted for 10 min in a Hidex 600 SL. Concentrations were calculated using linear regression of the counts per minute obtained from a standard curve of known concentrations.

### Mass Spectrometry

#### MALDI-MS

For the matrix-assisted laser desorption/ionization mass spectrometry (MALDI-MS) analysis, 1 μL of each protein sample (“idle”, “stirring”, and “stock”) was diluted with 2 μL of 0.1% trifluoroacetic acid (TFA) and then 1 μL of this diluted protein solution was mixed with 0.5 μL matrix solution, consisting of 5 mg/mL α -cyano-4-hydroxy cinnamic acid, 80% acetonitrile, 0.1% TFA, and added to a MALDI stainless steel plate. MS and MS/MS spectra were acquired using an Autoflex Speed MALDI TOF/TOF mass spectrometer (Bruker Daltonics, Bremen, Germany) in positive reflector and linear mode. All spectra were externally calibrated using Peptide calibration standard II (Bruker Daltonics) for reflector mode and Protein calibration standard I (Bruker Daltonics) for the linear mode.

#### Peak Identification in HPLC-UV by MS

For identification of which peak in the HPLC-UV runs corresponds to the intact tau fragment, the connected quadrupole (LCMS-2020, Shimadzu) was run in scan mode. During the absorbance peak at 3.6 min, the 8 most prominent *m*/*z* peaks detected was consistent with different charge states of the theoretical molecular weight of Tau304–380C322S, i.e. 8379.4 Da (Figure S4). The mass spectrometer was operated in ESI+ mode with a 350 °C interface temperature, 250 °C DL temperature, 200 °C heat block temperature, 1.5 L N_2_ per minute as nebulizing gas, 15 L N_2_/min as drying gas, and an interface voltage of 4.5 kV.

#### LC-MS/MS

For the LC-MS/MS analysis, all three protein samples were digested by adding sequencing-grade modified trypsin (Promega, Madison, WI, USA) to a protease:protein ratio of around 1:50 and incubated at 37 °C for 4 h. Then trypsin was added again to a final protease:protein ratio of around 1:25 and the samples were incubated overnight at 37 °C. The next day, formic acid (FA) was added to a final concentration of 0.5%. The peptides were cleaned up by C18 reversed-phase micro columns using an 2% acetonitrile (ACN), 0.1% FA equilibration buffer, and an 80% ACN, 0.1% FA elution buffer. The collected peptide samples were dried in a fume hood and resuspended in 15 μL 2% ACN, 0.1% FA before analysis on the high-resolution MS. The peptide samples were analyzed with LC-MS/MS by injecting them on to an ultrahigh pressure nanoflow chromatography system (nanoElute, Bruker Daltonics). The peptides were loaded onto an Acclaim PepMap C18 (5 mm, 300 μm i.d., 5 μm particle diameter, 100 Å pore size) trap column (Thermo Fisher Scientific) and separated on a Bruker Pepsep Ten C18 (75 μm× 10 cm, 1.9 μm particle size) analytical column (Bruker Daltonics). Mobile phase A (2% ACN, 0.1% FA) was used with the mobile phase B (0.1% FA in ACN) for 45 min to create a gradient (from 2 to 17% B in 20 min, from 17 to 34% B in 10 min, from 34 to 95% B in 3 min, at 95%B for 12 min) at a flow rate of 400 nL/min and a column oven temperature of 50 °C. The peptides were analyzed on a quadrupole time-of-flight mass spectrometer (timsTOF Pro, Bruker Daltonics), via a nano electrospray ion source (Captive Spray Source, Bruker Daltonics) in positive mode, controlled by the OtofControl 5.1 software (Bruker Daltonics). The temperature of the ion transfer capillary was 180 °C. A DDA method was used to select precursor ions for fragmentation with one TIMS-MS scan and 10 PASEF MS/MS scans. The TIMS-MS scan was acquired between 0.60–1.6 V s/cm^2^ and 100–1700 *m*/*z* with a ramp time of 100 ms. The 10 PASEF scans contained maximum of 10 MS/MS scans per PASEF scan with a collision energy of 10 eV. Precursors with maximum 5 charges with intensity threshold to 5000 au and a dynamic exclusion of 0.4 s were used. Raw data from the LC-MS/MS were processed using Mascot Distiller (version 2.8.5) and searched using Mascot Daemon (version 2.8) against an in-house database containing the sequence for tau304–380C322S fragment with the following settings; precursor ion tolerance: 10 ppm, MS/MS fragment mass tolerance: 0.015 Da, protease: None, Variable Modifications: Acetyl (Protein N-term), Deamidated (NQ), and Oxidation (M). When using None as a setting for the protease, Mascot will search each protein sequence without any enzyme cleavage specificity, not only look for the tryptic peptides cleaved C-terminally of arginine and lysine. If the tau fragment is cleaved at any another amino acid this will be shown in this database search. Peptides were considered identified if the individual ion score were greater than 34 (*p* < 0.005) and the peptide was identified at least two times within the data set.

## Supplementary Material



## Notes

HPLC chromatograms, SDS-PAGE analyses, HPLC-UV-MS and MALDI-TOF-MS, MALDI-TOF MS/MS identification of cleavage sites, investigations of peptide degradation during prolonged solubility incubations with and without stirring at 37 °C, and a LSC standard curve used for quantification (PDF)
