# Supplementary material for: Solubility and Metastability of the Amyloidogenic Core of Tau
Source: ACS Chem Neurosci. 2026 Jan 23;17(3):592–8. doi: 10.1021/acschemneuro.5c00784 (PMC12879728; doi:10.1021/acschemneuro.5c00784)
Supplement: Supplementary file 1 [file cn5c00784_si_001.pdf]

Supporting Information for: On the Solubility and Metastability of the Amyloidogenic Core of Tau

Emil Axell,[†] Andreas Carlsson,[†] Max Lindberg,[†] Katja Bernfur,[†] Emma Sparr,[‡] and Sara Linse[*,†]

†*Biochemistry and Structural Biology, Lund University, SE-221 00 Lund, Sweden*

‡*Division of Physical Chemistry, Department of Chemistry, SE-221 00 Lund, Sweden*

E-mail: emil.axell@biochemistry.lu.se

The importance of stirring during fibril formation

Shear forces and mixing have been established as important parameters that influence the aggregation kinetics of amyloid proteins.[1,2] To investigate how mixing affects tau solubility measurements, 7 μM monomeric tau was incubated at 37 °C without (idle) and with stirring using a magnetic stir bar (experimental setup as given in Figure S1(a)). After 240 h, the samples were divided into new tubes, either to start stirring in the idle sample or to stop stirring in the stirred sample. In addition, seeds from the stirred sample were added to the idle sample at 1 % mass concentration to discriminate between the effect of stirring and the presence of preformed fibrils. Aliquots of each sample were frequently collected for analysis over time. A part of the collected aliquot was frozen for simultaneous analysis by SDS-PAGE (Figure S2(a)), and the rest was separated from the fibrils by filtration. The tau concentrations in the filtrates were quantified using HPLC with UV absorbance in the

same manner as described in Figure 1, and are shown as a function of time in Figure S1(b). Typical chromatograms are shown in (c) (idle) and (d) (stirring). The same filtrates were analyzed by SDS-PAGE (Figure S2(b)).

It is evident that stirring dramatically accelerates aggregation, both with stirring from the beginning (blue squares) and when stirring was initiated in a sample that had been standing idle for 240 h without significant aggregation (purple triangles). The plateau concentration reached in the stirred sample (8.3 ± 3.8 nM) is in good agreement with the solubility obtained in Figure 1(a) (6.3 ± 3.5 nM), indicating that the measurements are robust and independent of the separation method (filtration vs centrifugation). Furthermore, several peaks appear with increasing intensity over time in the chromatogram of the idle sample (c), with no m/z ratios in the mass spectrum matching intact peptide. This, in combination with the weak but prominent shadow that appears below the monomeric bands in SDS-PAGE (figure S2(a) and (b)), suggests that the peptide in its non-aggregated state is subject to chemical changes such as truncations and/or chemical modifications. LC-MS/MS analysis on a high-resolution mass spectrometer of trypsin-digested tau from late time points (600 h) of the idle sample (Figure S8) reveals at least six cleavage sites. Analysis by matrix-assisted laser desorption/ionization time of flight mass spectrometry (MALDI-TOF MS) (figures S5-S6) also reveal multiple masses appearing and evolving over time in the mass spectrum, which cannot be assigned to the intact tau peptide. Additional MS/MS analysis (figure S7) identifies one of the most prominent cleavage sites as located 12 residues from the N-terminus.

It can be noted that the monomer concentration in the "idle then stirred" sample does not reach the same plateau as that of the sample stirred from the beginning. A plausible explanation may be that fibrils of modified monomer form over time, or a heterogeneous fibril consisting of a mixture of intact tau along with partially degraded variants. This could result in fibrils with altered thermodynamic properties, e.g. a higher chemical potential compared

to pure fibrils, leading to a higher observed apparent monomer concentration. This apparent solubility does not represent the "true" solubility of the intact peptide. This can only be approached when the monomers form the most stable aggregate possible.

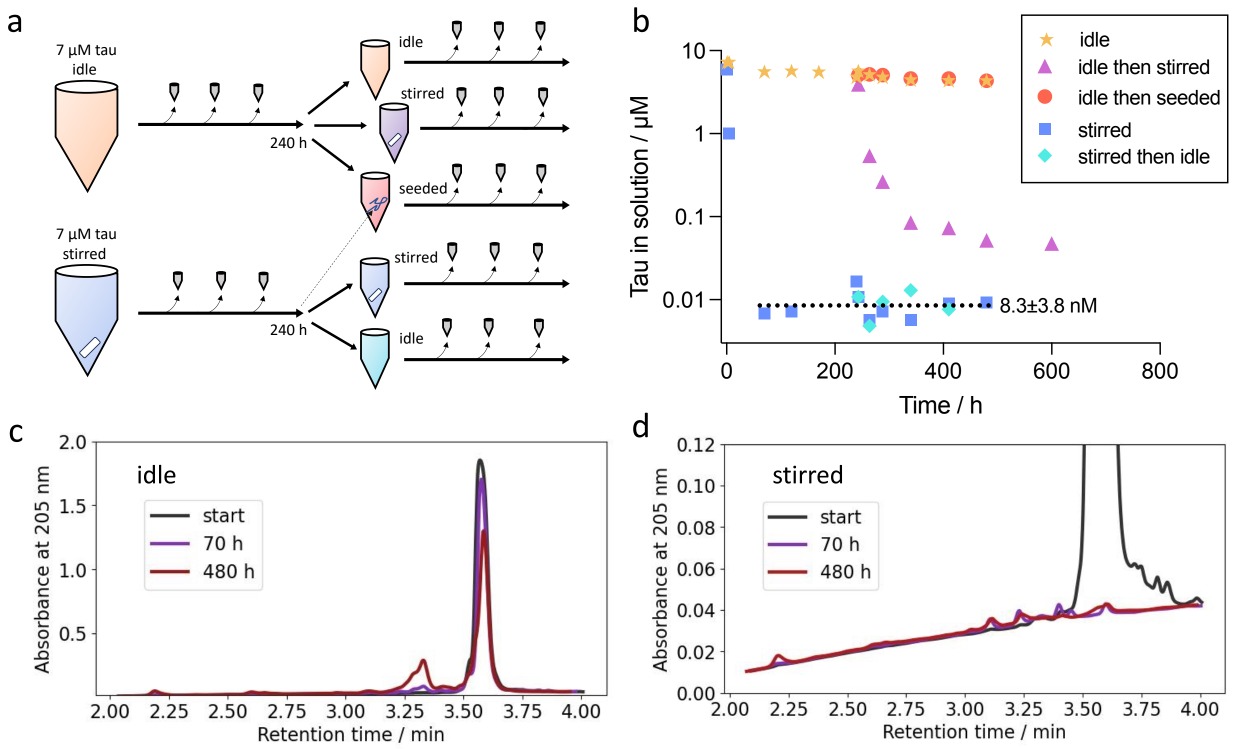

Figure S1: Investigation of the effect of mixing on the tau solubility measurements. (a) Experimental setup. 7 µM monomeric tau was incubated at 37 °C, one sample at idle conditions and one with stirring. As a function of time, fractions of the samples were removed for analyses. After 240 h the idle sample was divided into three samples: one kept idle, one for which stirring was started, and one supplemented with seeds. Also, the stirred sample was divided into two: one kept stirring and one made idle. (b) Quantification of the monomeric tau using HPLC UV-absorbance, for the samples with symbols as given in the legend. The mean value of the concentrations of the 9 last time points in the sample stirred from the beginning is 8.3 ± 3.8 nM. (c and d) HPLC chromatograms, absorbance at 205 nm, for idle (c) and stirred (d) samples. The peak eluting at 3.6 min was identified using mass spectrometry as tau and hence the area of this peak was used to generate the plot in (b). Note the zoomed-in y-axis in (d), to identify the very small tau peak close to equilibrium at 70 and 480 h.

Several factors may contribute to the slow kinetics of tau aggregation under idle conditions. In a non-agitated liquid, Brownian motion is the limiting factor for peptides to come into close proximity of each other. This, combined with the effect of shear forces on

the primary and secondary nucleation rate,[1] can help explain why, even with seeds, the idle samples do not aggregate significantly within the experimental time frame. Some level of applied mixing appears advantageous for the precipitation of a supersaturated peptide solution and to help ensure that equilibrium is reached. The sample that was first stirred and then kept idle after fibrils had formed was analyzed by SDS-PAGE (Figure S2a) without prior removal of fibrils. Thus, the intensity of the monomer band represents the total concentration of tau in dispersion, both aggregated and monomeric forms.

The gradual disappearance of tau can be attributed to the sedimentation of the fibrils. As fibrils disappear from the solution, they can no longer catalyze the formation of new fibrils through secondary nucleation or consume monomers via elongation at the same rate as when they are in suspension. As a result, the system relies predominantly on primary nucleation to reach equilibrium by consuming the remaining monomer. The much higher nucleation energy barrier of primary nucleation compared to secondary nucleation and elongation[3,4] results in slow kinetics and a large and long-lived metastable zone. The observation that simply allowing a tube of tau fibrils to stand still on the bench for 48 h was sufficient for most fibrils to sediment was unexpected, as slightly longer fragments of tau have been reported to require ultracentrifugation for efficient sedimentation.[5,6] However, the studies differ in tau sequence and buffer conditions, factors that may substantially alter sedimentation behavior by affecting electrostatic interactions and fibril structure.

The discovery of a high sedimentation tendency led us to attempt to separate monomers from fibrils using a regular bench top centrifuge (20000xg). This was sufficient for pelleting the fibrils in our samples.

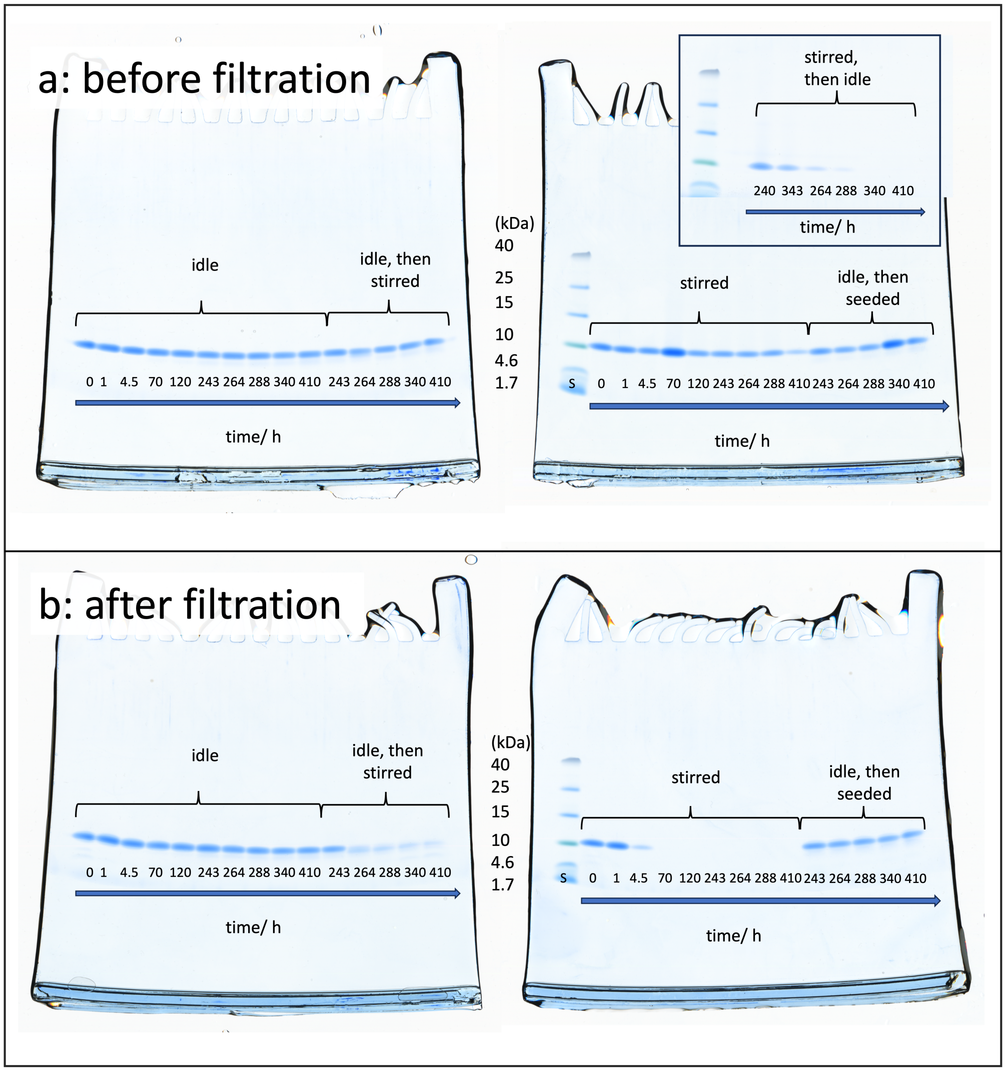

Figure S2: SDS-PAGE of the samples from Figure S1. The number below each protein band indicates the time (hours) since starting the reaction. Note that the tau aggregates dissolve into monomeric species in the SDS loading buffer, making the band for monomeric tau at $\sim$ 10 kDa represent the total concentration tau in the loaded sample. (a) Before filtration - everything in dispersion is seen in the gels. The insert to the right shows the sample for which stirring was stopped after 240 h. The gradual decrease in band intensity is interpreted as sedimentation of fibrils. (b) After filtration - only species smaller than the pore size of 200 nm pass the filter and are seen in the gels.

Comparison of "idle" and "idle then stirred" HPLC UV chromatograms

The chromatograms obtained after incubation of tau at 37 °C show the emergence of distinct species with retention times other than of intact tau (Figure S3). The formed species are different under idle conditions compared to stirring or to idle then stirred samples.

At least three peaks (at 2.6, 3.15, 3.3 min) grow much faster when stirring is introduced in the initial idle condition. The near disappearance of the monomer peak (3.6 min) is explained by aggregation into fibrils. The smaller peak at 3.3 min in the idle then stirred sample may indicate that this species is further changed upon stirring (e.g. cleaved into smaller parts), it could itself be prone to aggregation or may be incorporated into fibrils.

A speculative, albeit plausible, explanation for the additional modifications observed in the initially idle, then stirred sample is that the fibril surface could catalyze chemical reactions of the monomers. Fibril-mediated catalysis is an emerging topic, and amyloid fibrils of several proteins have recently been shown to catalyze the hydrolysis and chemical modification of bound substrates.[7–9]

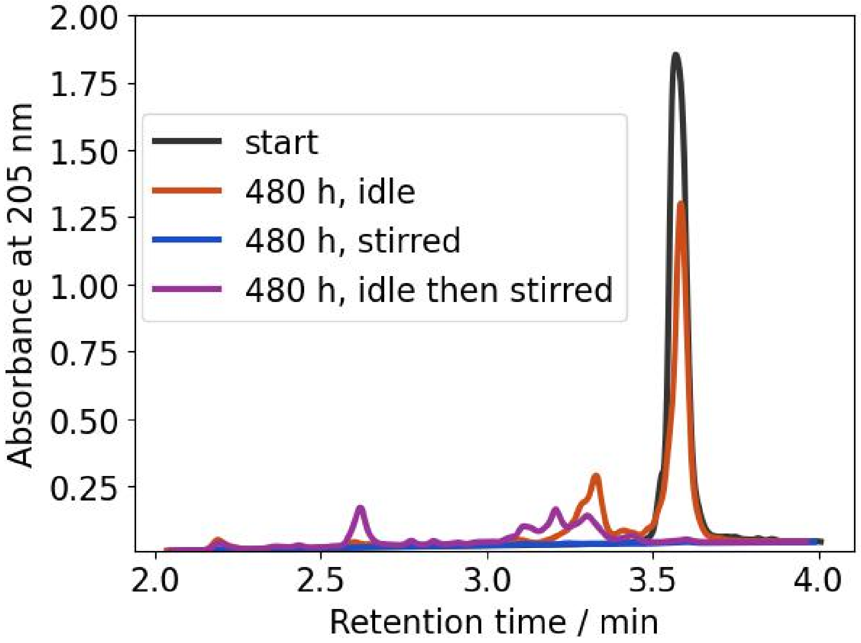

Figure S3: Comparison of chromatograms of UV absorbance of samples from Figure S1. The "idle then stirred" sample is made from the idle sample, by dividing the idle sample in two and start stirring after 240 h.

Mass spectrometry

To further investigate the identity of the emerging peaks in the HPLC chromatograms during prolonged incubation of the tau peptide (Figure S3), a more thorough investigation was performed using mass spectrometry (MS).

The theoretical mass of intact tau304-380C322S fragment without the N-terminal methionine and with an acetylated N-terminal is 8379.42 Da. Intact MALDI-MS analysis was performed to get information about the protein content in the samples at the end time of the experiment. Spectra were acquired in positive linear mode at a mass range of m/z 2800 to 11000 (figure S5). This m/z range was chosen to ensure that the presence of any contaminating proteins larger than tau could be excluded and that potential degradation products would be detected as lower mass peaks. The "stock" sample (Figure S5, top spectrum) is shown to be highly pure and mainly contains the signals for the 1+ and 2+ charged intact tau sequence (signals with m/z 8381.288 and 4188.302, respectively). These low charge states

are typical for peptides of this size and represent the only prominent signals observed in the spectrum.

When analyzed by HPLC-UV-MS (figure S4), the stock elutes as a single UV peak at 3.6 min, with masses corresponding to intact tau in multiple protonation states due to ionization.

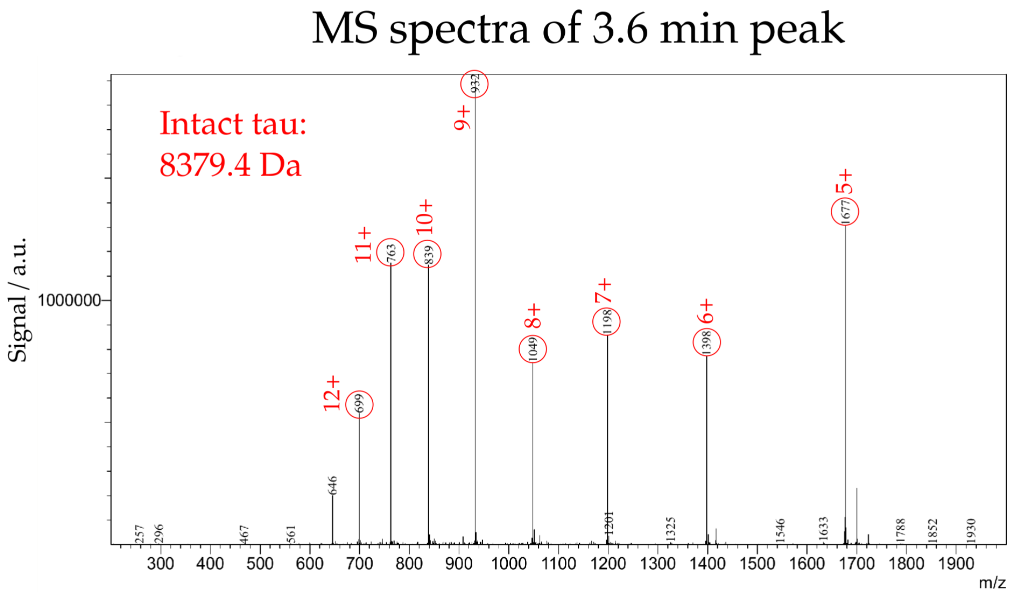

Figure S4: The average spectrum from t_R 3.567 to 3.600 min, corresponding to the center of the suspected tau peak, shows prominent ions consistent with intact tau (8379.42 Da). Charge states vary from n=5-12 and match the expected relation $m/z * n - n = 8379$ (within detector accuracy) where n is the number of extra hydrogens.

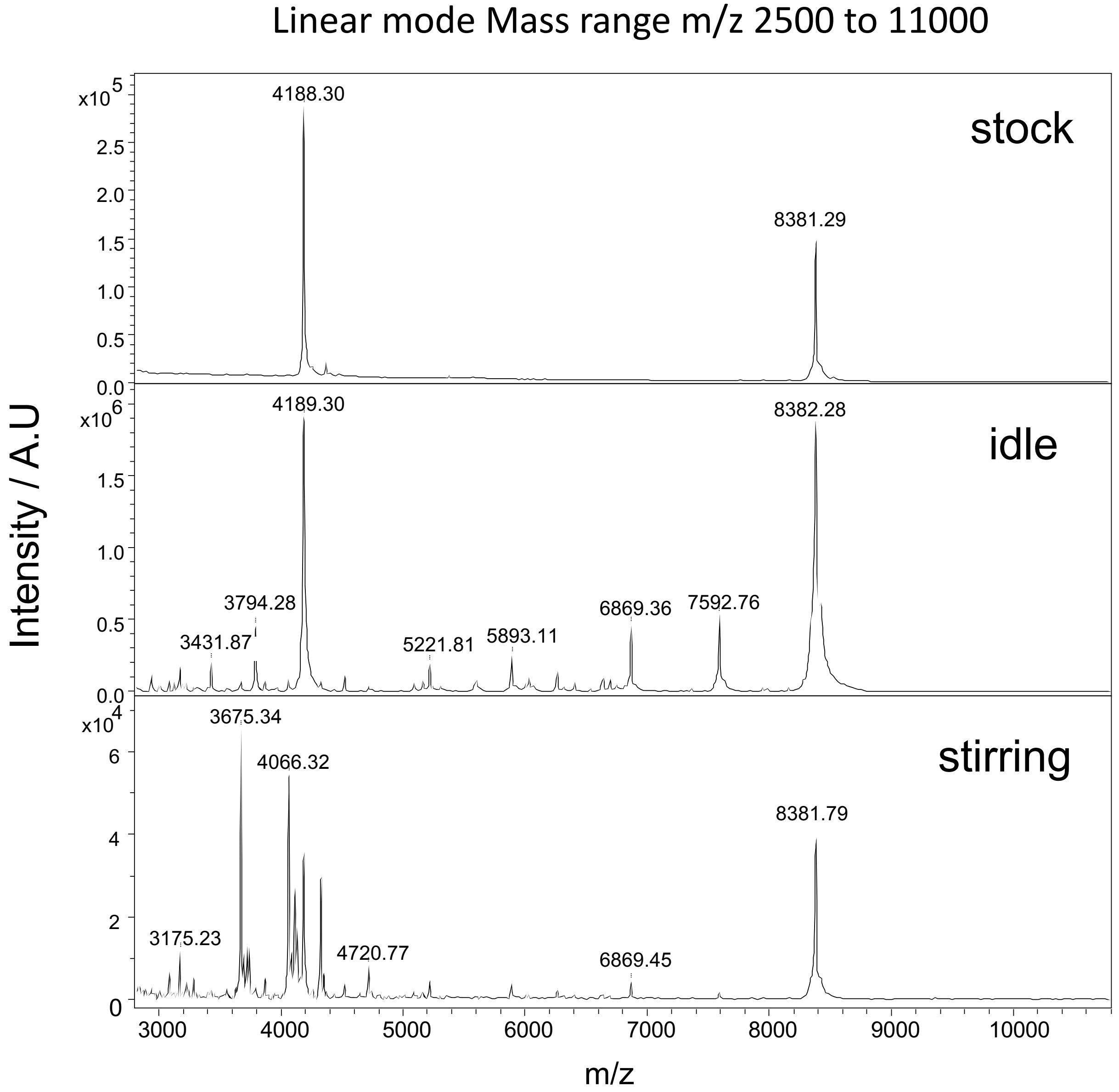

Figure S5: Intact MALDI MS spectra in linear mode of the tau samples ”stock” (control sample), ”idle”, and “”stirring”. The start concentration of monomeric tau was 7 µM and the ”idle” and ”stirring” samples were then collected after 600 h for this ms analysis.

These correspond well to the theoretical mass of the tau fragment. On the contrary, a lot of signals with a lower mass than the intact tau fragment were detected in both the ”idle” and ”stirred” samples (figure S5, middle and lower spectrum, respectively). Some of the signals are present in both samples and some are only detected in one or the other. In the sample ”idle” as many as 21 signals were detected clearly showing a breakdown of tau.

It should be noted that the overall concentration in the “”stirred” sample is much lower than the other two due to aggregation. To investigate the presence of even lower mass tau fragments, MALDI spectra were then acquired in positive reflector mode at a mass range of m/z 900 to 4300 (Figure S6).

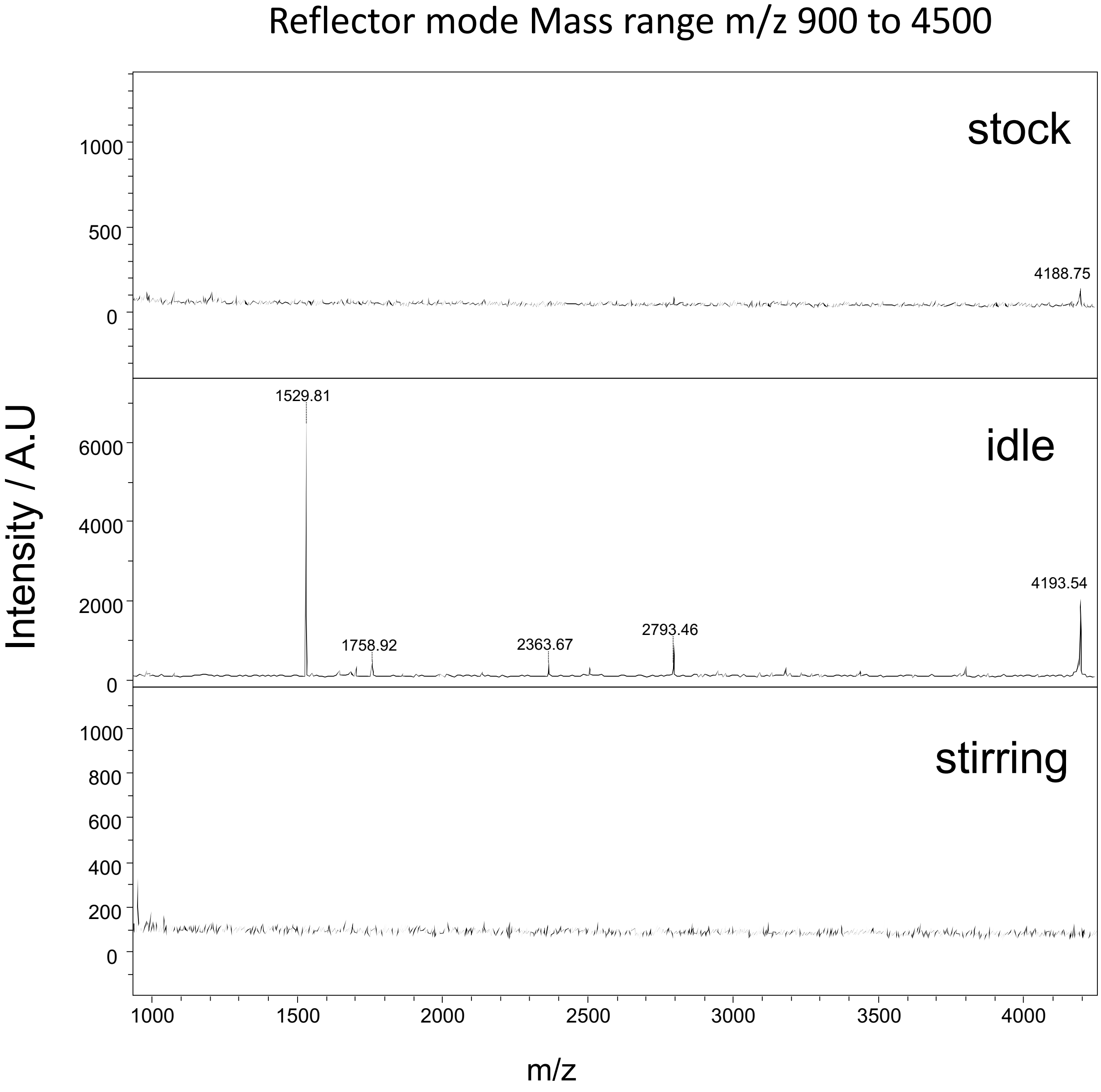

Figure S6: MALDI MS spectra in reflector mode of the tau samples ”stock” (control sample), ”idle”, and “”stirring”, same as in figure S5. The start concentration of monomeric tau was 7 µM and the ”idle” and ”stirring” samples were then collected after 600 h for this ms analysis

No signals were detected in the "stock" or "stirred" sample at this mass range, but in the "idle" sample several signals were visible. The most intense signal at m/z 1529.81 (figure S6) was further analyzed with MALDI-MS/MS (Figure S7) and identified as a peptide consisting of the 12 most C-terminal residues of 304-380 tau, cleaved between N368 and K369 relative 441 numbering.

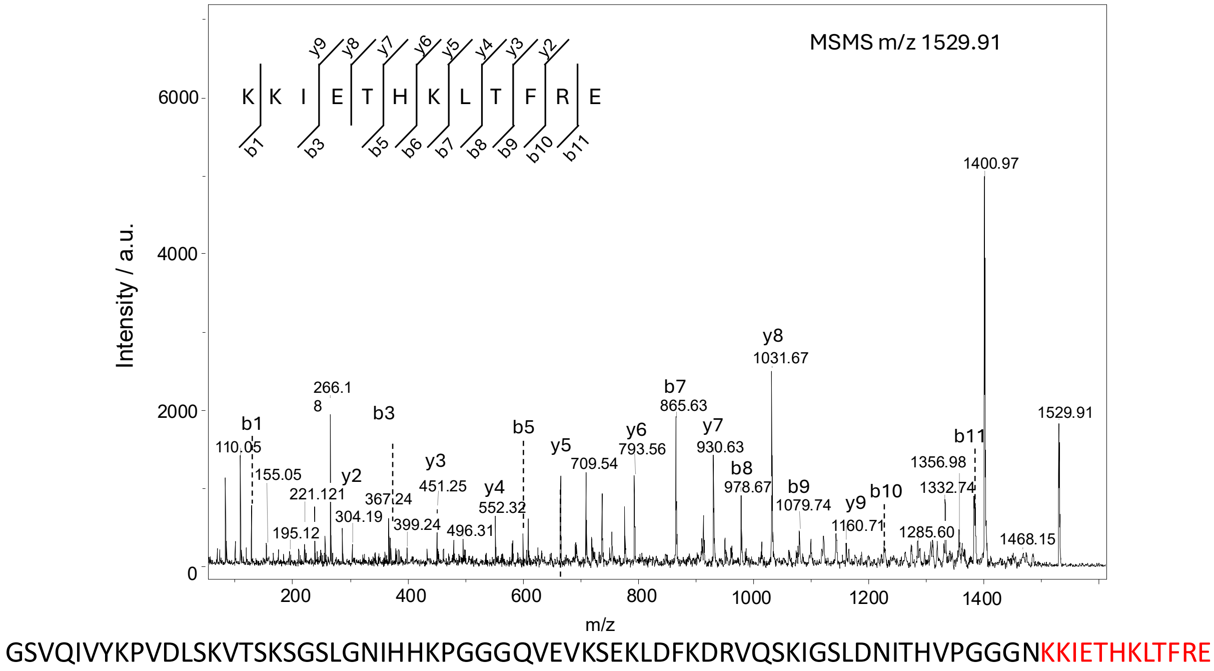

Figure S7: MALDI MS/MS of the most prominent proteolytic cleavage product under idle conditions (1529 m/z S6) confirms that the cleavage site is located 12 residues from the C-terminus. In the top left corner, the sequence of the peptide is shown where the detected ions are presented. The signals corresponding to the identified b- and y-ions are marked in the spectrum. The full sequence is shown at the bottom, with the 1529 Da fragment colored in red.

We conclude that the tau "stock" sample is pure and contains mainly one sequence. Over time, additional smaller species appear, notably under "idle" conditions. These emerging peaks in the HPLC chromatogram likely reflect degradation products such as truncations, although we cannot rule out that other chemical modifications also occur. Further analyses, such as tandem-MS or peptide mapping would be required to more definitively determine whether they represent truncations, chemical modifications, or other intermediates.

These degradation products arise after prolonged incubation. We therefore interpret them as chemical artifacts rather than physiological relevant alterations of the tau peptide.

Tryptic digests of the peptide samples were further analyzed with LC-MS/MS on a high-resolution MS (figure S8). The detected cleavage sites that do not correspond to tryptic cleavage sites are marked with red scissors. The red number at each cleavage site corresponds to the number of times these non-tryptic cleavage site were detected, which is a semi-quantitative measure of the frequency of the cleavage. It is evident that the "idle" sample is more cleaved than the stirred sample and notably, the most prominent cleavage site, between N368 and K369 is detected 9 times. It is the same cleavage site as for the most intense peptide signal in the MALDI-MS reflector mode analysis and also the only non-tryptic cleavage site that were detected in the "stock" sample.

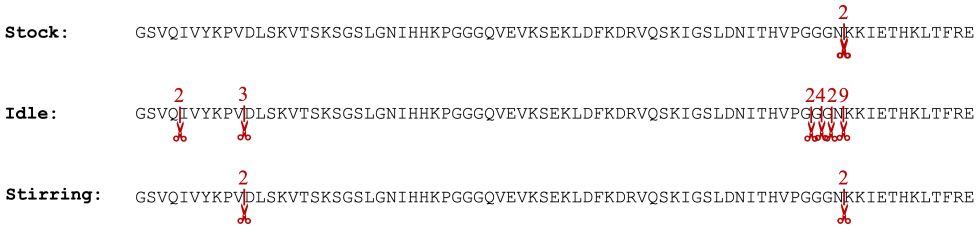

Figure S8: Identified non-tryptic cleaving sites, determined by LC-MS/MS, are marked with red scissors and the number of times a peptide produced at that cleavage site was detected can be seen as a red number next to each site.

Standard curve used for LSC

Known concentrations of radiolabled tau were serially diluted to generate a standard curve used for quantification (figure S9).

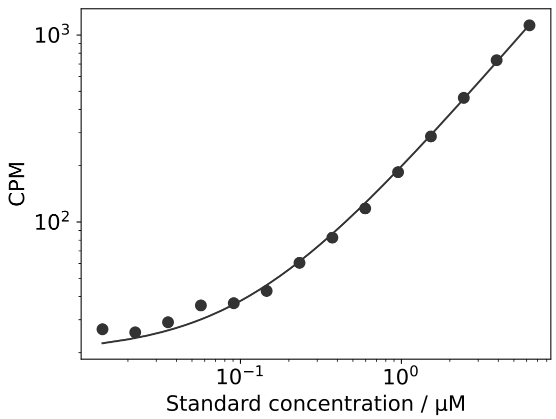

Figure S9: Standard curve obtained by LSC with a linear regression. Raw LSC data are expressed as counts per minute (CPM).

References

(1) Axell, E.; Hu, J.; Lindberg, M.; Dear, A. J.; Ortigosa-Pascual, L.; Andrzejewska, E. A.; Šneiderienė, G.; Thacker, D.; Knowles, T. P.; Sparr, E., et al. The role of shear forces in primary and secondary nucleation of amyloid fibrils. *Proceedings of the National Academy of Sciences* **2024**, *121*, e2322572121.

(2) Grigolato, F.; Colombo, C.; Ferrari, R.; Rezabkova, L.; Arosio, P. Mechanistic origin of the combined effect of surfaces and mechanical agitation on amyloid formation. *ACS nano* **2017**, *11*, 11358–11367.

(3) Rodriguez Camargo, D. C.; Sileikis, E.; Chia, S.; Axell, E.; Bernfur, K.; Cataldi, R. L.; Cohen, S. I.; Meisl, G.; Habchi, J.; Knowles, T. P., et al. Proliferation of tau 304–380 fragment aggregates through autocatalytic secondary nucleation. *ACS Chemical Neuroscience* **2021**, *12*, 4406–4415.

(4) Cohen, S. I.; Cukalevski, R.; Michaels, T. C.; Šarić, A.; Törnquist, M.; Vendruscolo, M.; Dobson, C. M.; Buell, A. K.; Knowles, T. P.; Linse, S. Distinct thermodynamic signatures of oligomer generation in the aggregation of the amyloid-β peptide. *Nature chemistry* **2018**, *10*, 523–531.

(5) Lövestam, S.; Li, D.; Wagstaff, J. L.; Kotecha, A.; Kimanius, D.; McLaughlin, S. H.; Murzin, A. G.; Freund, S. M.; Goedert, M.; Scheres, S. H. Disease-specific tau filaments assemble via polymorphic intermediates. *Nature* **2024**, *625*, 119–125.

(6) Weismiller, H. A.; Murphy, R.; Wei, G.; Ma, B.; Nussinov, R.; Margittai, M. Structural disorder in four-repeat Tau fibrils reveals a new mechanism for barriers to cross-seeding of Tau isoforms. *Journal of Biological Chemistry* **2018**, *293*, 17336–17348.

(7) Wolfram, M.; Tiwari, M. K.; Hassenkam, T.; Li, M.; Bjerrum, M. J.; Meldal, M. Cascade autohydrolysis of Alzheimer's Aβ peptides. *Chemical Science* **2023**, *14*, 4986–4996.

(8) Horvath, I.; Wittung-Stafshede, P. Amyloid Fibers of α-Synuclein Catalyze Chemical Reactions. *ACS Chemical Neuroscience* **2023**, *14*, 603–608.

(9) Frey, L.; Buratti, F. A.; Horvath, I.; Parate, S.; Kumar, R.; Riek, R.; Wittung-Stafshede, P. ATP Hydrolysis by α-Synuclein Amyloids is Mediated by Enclosing β-Strand. *Advanced Science* e08441.
